# Analysis of intra-annual mortality fluctuations by cause of death in Italy, 2004–19

**DOI:** 10.1093/eurpub/ckag165

**Published:** 2026-09-22

**Authors:** Isabella Marinetti, Dmitri A Jdanov, France Meslé, Domantas Jasilionis, Fanny Janssen

**Affiliations:** Laboratory of Demographic Data, Max Planck Institute for Demographic Research, Rostock, Germany; Population Research Centre, Faculty of Spatial Sciences, University of Groningen, Groningen, The Netherlands; Laboratory of Demographic Data, Max Planck Institute for Demographic Research, Rostock, Germany; Institut National d‘Études Démographiques (INED), Paris, France; Laboratory of Demographic Data, Max Planck Institute for Demographic Research, Rostock, Germany; Max Planck—University of Helsinki Center for Social Inequalities in Population Health, Rostock, Germany, Finland; Demographic Research Centre, Vytautas Magnus University, Kaunas, Lithuania; Netherlands Interdisciplinary Demographic Institute, KNAW/University of Groningen, The Hague, The Netherlands

## Abstract

Seasonal fluctuations in mortality significantly affect population health and remain an important public health challenge, particularly in the context of climate change and population ageing. Evidence on how different causes of death shape seasonal mortality patterns remains limited. We apply a novel framework to quantify, in a systematic, transparent, and comparable manner, the absolute and relative contributions of 17 cause-of-death groups to seasonal mortality in Italy. We analyzed monthly mortality data from the Italian National Institute of Statistics, disaggregated by cause of death, sex, and 5-year age groups, 2004–19. Excess mortality for 17 major causes of death was quantified by comparing the observed age-standardized death rate (SDR) with the baseline SDR, defined as the average of the 3 months with the lowest mortality each year. Heart and respiratory diseases were the leading contributors to excess mortality, accounting for approximately 40% and 15% of the intra-annual burden, respectively. The summer months exhibited significantly smaller excess mortality. Respiratory diseases showed the highest relative impact compared to the other causes of death. While baseline mortality decreased over time, the relative intra-annual burden remained stable or increased for specific causes, indicating persistent or growing seasonal vulnerabilities. Intra-annual mortality fluctuations in Italy were mainly driven by cardiovascular and respiratory diseases and have remained stable over time despite improvements in overall mortality. These persistent patterns highlight unmet seasonal vulnerabilities and the importance of integrating cause-specific seasonality into health planning, particularly in light of ageing populations and climate change challenges.

## Introduction

Mortality patterns are often influenced by seasonal variations, particularly in temperate regions, where death rates tend to peak, predominantly, in the winter and, to a lesser extent, during summer heatwaves [1–3]. These intra-annual fluctuations not only reflect environmental and social stressors, such as cold temperatures, extreme heat, or infectious disease spread but also healthcare system capacity and public health planning [3, 4]. Moreover, these seasonal variations contribute to short-term changes in overall mortality and can influence life expectancy levels [5]. When analyzing all-cause seasonal mortality patterns, we are essentially observing multiple, heterogeneous cause-specific processes operating simultaneously. Therefore, understanding the underlying patterns in cause-specific mortality is essential to accurately identifying the mechanisms through which these fluctuations affect overall mortality and life expectancy. However, while seasonality itself is well documented, an analytical and systematic approach to estimate the main cause-of-death drivers of mortality within the year is still lacking.

Indeed, specific causes of death respond differently to seasonal risks. Respiratory diseases, such as influenza and pneumonia, typically peak in winter due to cold temperatures and the spread of seasonal viruses [6, 7]. Cardiovascular diseases also show seasonal variation, with increased risks during both cold spells and heatwaves [8, 9]. In contrast, neoplasms tend to exhibit little to no seasonality, showing stable mortality rates throughout the year [10], but the results may depend on the country analyzed [11]. External causes of death, such as accidents or suicides, display reverse seasonal trends, with some studies reporting peaks in spring or summer [12, 13]. These differences underscore the importance of analyzing seasonal mortality from a cause-specific perspective, as the timing, magnitude, and direction of fluctuations vary substantially across causes.

In this context, Italy is one of the European countries most affected by heat waves and flu epidemics in recent years [6, 14]. Life expectancy at birth showed several fluctuations attributable to short-term events, including the economic crisis in 2008–10 [15], heatwaves in 2003 and 2015 [16, 17], extreme winter temperatures in 2005, 2012, and 2017, and flu epidemics in 2015 and 2017 [7, 8, 18]. The few cause-specific studies in Italy suggest similar cause-specific patterns as observed in other European countries, with high excess mortality for cardiovascular and respiratory diseases [8, 19, 20], and peaks in external mortality during spring and summer [21, 22]. However, the mentioned studies have mainly focused on specific high-burden events (e.g. heatwaves or epidemics) or analyzed single causes of death separately. As a result, we still lack an integrated and systematic cause-specific picture of seasonal excess mortality in Italy.

In order to fill this gap, we propose a framework that applies the excess mortality approach. The excess mortality method has been commonly used to assess mortality impacts during exceptional mortality events, such as pandemics or flu epidemics. When applied to seasonal mortality, the approach not only allows to quantify the magnitude of excess mortality but also identifies which causes of death are primarily responsible for excess mortality during particular seasons.

In our study, we quantify, systematically and comparably, the absolute and relative contributions of 17 cause-of-death groups to seasonal mortality in Italy, during 2004–19, using high-quality national monthly mortality data. By doing so, we deepen the existing literature by attributing all-cause excess mortality to specific causes of death, uncovering how different causes contribute to the observed seasonal fluctuations and to what extent they shape overall mortality trends throughout the year.

## Methods

### Cause-of-Death data

We employed monthly death count data from the Italian National Institute of Statistics (ISTAT), disaggregated by cause of death (ICD-10), sex, and 5-year age groups from 0 to 90+, covering the period from 2004 to 2019. Annual population data by sex and age group were also obtained from ISTAT. We distinguished 17 major cause-of-death groups (Table S1 for the groups, the ICD10 codes, and their share in overall mortality). Cardiovascular diseases (56%), neoplasms (30%), and respiratory diseases (7%) contributed the most to mortality in Italy in 2004–19. See Appendix A, for more information on the cause-of-death selection and handling. We show the monthly unweighted average over 2004–19 in our main results. In the Supplementary Material, the time trend and specific-month results are available. Appendix B, provides the results by NUTS1 level (North-East, North-West, Centre, South, Islands). We decided to stop the analysis before the outbreak of COVID-19 to avoid a misinterpretation of the results.

### Methodological framework

To investigate seasonal patterns and quantify intra-annual excess mortality by cause of death, we applied an excess mortality-based framework commonly used to assess mortality impacts during high-mortality events, adapted here to seasonal mortality.

To understand whether the causes of death trends followed a seasonal pattern, we computed age-standardized death rates (SDR) using the 2013 European Standard Population [23], for each month, year, cause of death, and sex. Subsequently, to estimate the cause-specific excess deaths due to intra-annual fluctuations in mortality, we computed the baseline SDR (i.e. the counterfactual mortality rate in the absence of excess mortality), calculated as explained below. The baseline is defined separately for each cause of death, which allows seasonal deviations to be measured relative to the lowest observed mortality for that specific cause in each year (Fig. S2 in the Supplementary Material for the baseline months by cause and year).

For both the observed and baseline SDR, all deaths were first analyzed collectively (all causes combined) and then disaggregated by cause of death to evaluate their specific contributions to overall mortality dynamics. We computed both the absolute and relative contribution: while absolute excess deaths reveal the largest contributors to seasonal mortality in terms of volume, relative excess deaths highlight causes disproportionately affected by intra-annual variation.

#### Absolute impacts: cause-specific excess deaths due to intra-annual fluctuations in mortality

We conceptualized the baseline SDR as an average mortality rate based on the three months in each year with the lowest death counts by sex and cause of death, all ages combined. Using the 3 months with the lowest mortality allowed us to assess the counterfactual lowest observed level of mortality in a year, measuring intra-annual variation consistently over time. This was the lowest observed mortality level within each year and has the important advantages of overcoming stochastic fluctuations of mortality rates [5].


(1) $${\mathrm{SDR}}_{c,t}^{\mathrm{baseline}}=\sum_{x}m_{x,c,t}^{\mathrm{baseline}}\cdot w_{x}$$

where $w_{x}=\frac{P_{x}^{s}}{\sum_{x}P_{x}^{s}}$ are the population weights and $P_{x}^{s}$ is the European Standard Population 2013 at age *x*, while $m_{x,c,t}^{\mathrm{baseline}}$ is the counterfactual mortality rate at age *x,* cause of death *c*, and year *t*. The baseline and observed SDRs were computed for each cause of death independently, as well as for all-cause mortality (${\mathrm{SDR}}_{\mathrm{tot},t}^{\mathrm{baseline}}$), based on total mortality rates ($m_{x,\mathrm{tot},t}^{\mathrm{baseline}}$) and the same weights $w_{x}$. We have computed an additional analysis to check another possible baseline using median mortality within a year (Appendix C).

We quantified excess mortality as the difference between observed and baseline SDRs, for cause-specific mortality, sex, month, and year.


(2) $${\mathrm{SDR}}_{c,t,m}^{\mathrm{excess}}=\sum_{x}(m_{x,c,t,m}^{\mathrm{observed}}-m_{x,c,t}^{\mathrm{baseline}})\cdot w_{x}$$

where $w_{x}$ are the same as in (1) at age *x*, $m_{x,c,t,m}^{\mathrm{observed}}$ is the observed mortality rate at age *x,* cause of death *c,* year *t,* and month *m*, and $m_{x,c,t}^{\mathrm{baseline}}$ is the counterfactual mortality rate at age *x,* cause of death *c*, and year *t*. We have also computed it for all-cause mortality ($\mathrm{SD}R_{\mathrm{tot},t,m}^{\mathrm{excess}}$) similarly, just considering the overall mortality. Because the sum of cause-specific excess mortality may not equal total excess mortality, a multiplicative correction factor was applied. For each year, month, and sex, the ratio between total excess mortality and the sum of cause-specific excess mortality was computed:


(3) $$R_{c,t,m}=\frac{\mathrm{SD}R_{\mathrm{tot},t,m}^{\mathrm{excess}}}{\sum_{x}{\mathrm{SDR}}_{c,t,m}^{\mathrm{excess}}}$$

where $\mathrm{SD}R_{\mathrm{tot},t,m}^{\mathrm{excess}}\;$and $\mathrm{SD}R_{c,t,m}^{\mathrm{excess}}$ are computed as in (2). Therefore, corrected cause-specific excess deaths were then defined as:


(4) $${\mathrm{SDR}}_{c,t,m}^{\mathrm{excess},\;\mathrm{corr}}=R_{c,t,m}\cdot {\mathrm{SDR}}_{c,t,m}^{\mathrm{excess}}$$

We computed confidence intervals for the cause-specific and all-cause excess mortality estimates [24] (Appendix D).

Finally, we compared the excess mortality values by cause of death to the overall all-cause excess mortality. In order to do so, we computed a percentage contribution calculated as the ratio between the cause-specific and the all-cause excess mortality in that month. We also present the values for only winter and summer months as their monthly sum.

#### Relative impacts: cause-specific excess mortality ratio due to intra-annual fluctuations in mortality

Lastly, to understand which causes of death were relatively more affected by excess mortality, we computed the difference between observed (Fig. S1) and baseline (Fig. S2) SDR divided by the baseline SDR for each cause of death, sex, month, and year. This indicator allowed us to compare how much each cause was affected by seasonality, relative to its own baseline level, revealing causes particularly vulnerable to short-term mortality shocks. Values above 0 indicate that observed mortality exceeded the baseline for that cause, whereas values below 0 indicate mortality below the expected level.


(5) $$\mathrm{Relative}\;\mathrm{Excess}\;\mathrm{Death}s_{c,t,m}=\frac{{\mathrm{SDR}}_{c,t,m}^{\mathrm{observed}}-{\mathrm{SDR}}_{c,t,m}^{\mathrm{baseline}}}{{\mathrm{SDR}}_{c,t,m}^{\mathrm{baseline}}}$$

The analyses were conducted in R version 4.2.3.

## Results

### Intra-annual fluctuations of causes of death in Italy

**Figure 1:**
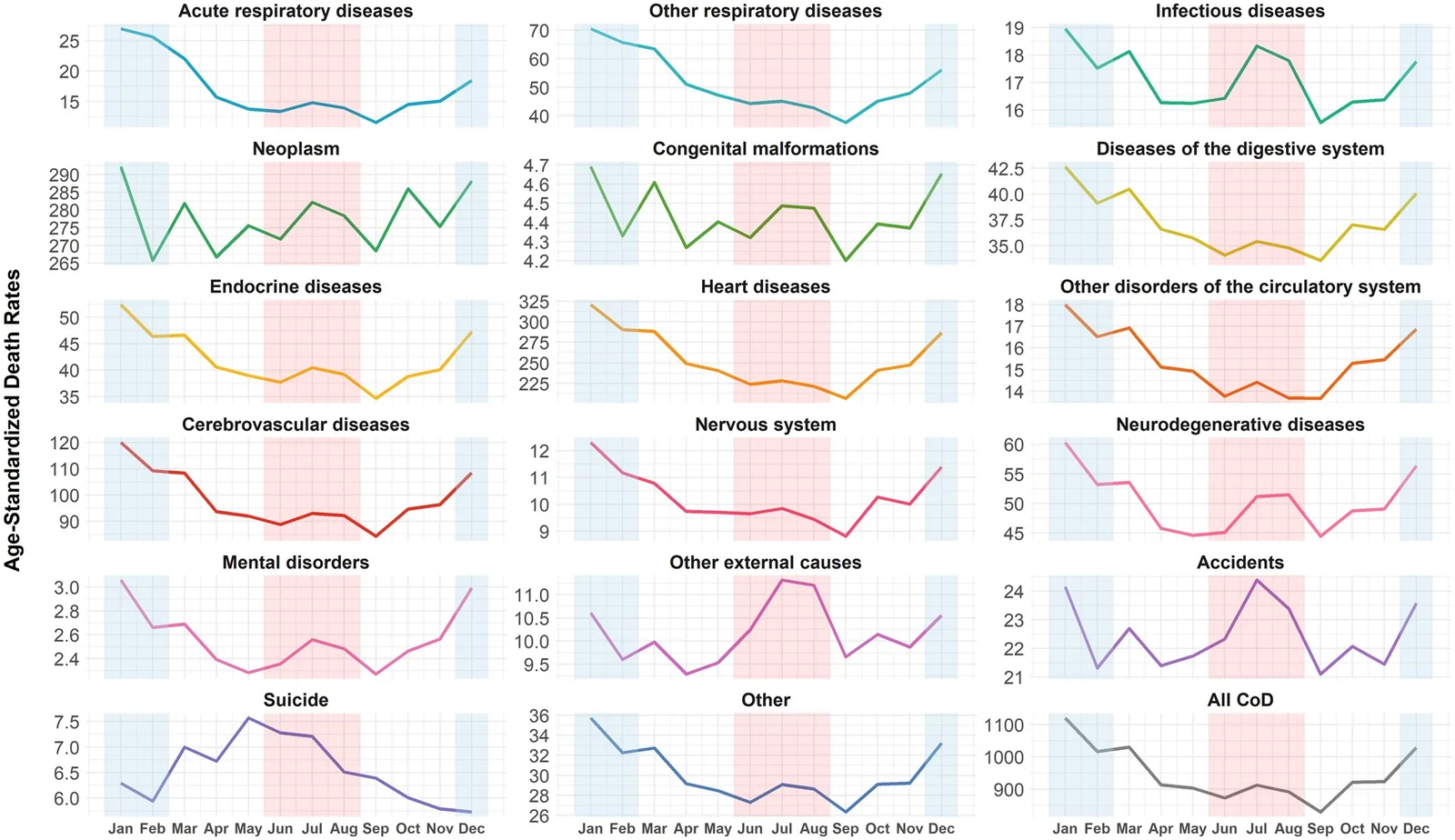
Age-SDRs (per 100.000) by month (blue shadow: winter, red shadow: summer) and cause of death, total Italian population, average 2004–19.

All-cause mortality followed a statistically significant seasonal pattern, as did the majority of cause-specific mortality rates (Fig. 1). Both all-cause mortality and most causes of death displayed marked peaks during the winter months (blue shadow), particularly those related to circulatory and respiratory conditions, with slight increases also during summer months (red shadow). In contrast, deaths due to suicide, other external causes, and accidents showed a reversed seasonal pattern, with peaks during summer months and lower mortality rates during colder months. Notably, congenital malformations and cancer did not exhibit any consistent seasonal pattern. The analysis at the NUTS1 level showed similar results (Fig. S7); all territories followed a similar seasonal pattern compared to the whole country, however, with different magnitudes. The South and Insular regions, which have the highest annual [25] and seasonal mortality [26], are confirmed to have the highest values in major causes of death, such as cardiovascular disease (heart and cerebrovascular) and respiratory diseases.

### Absolute impact of causes of death on excess all-cause mortality

**Figure 2:**
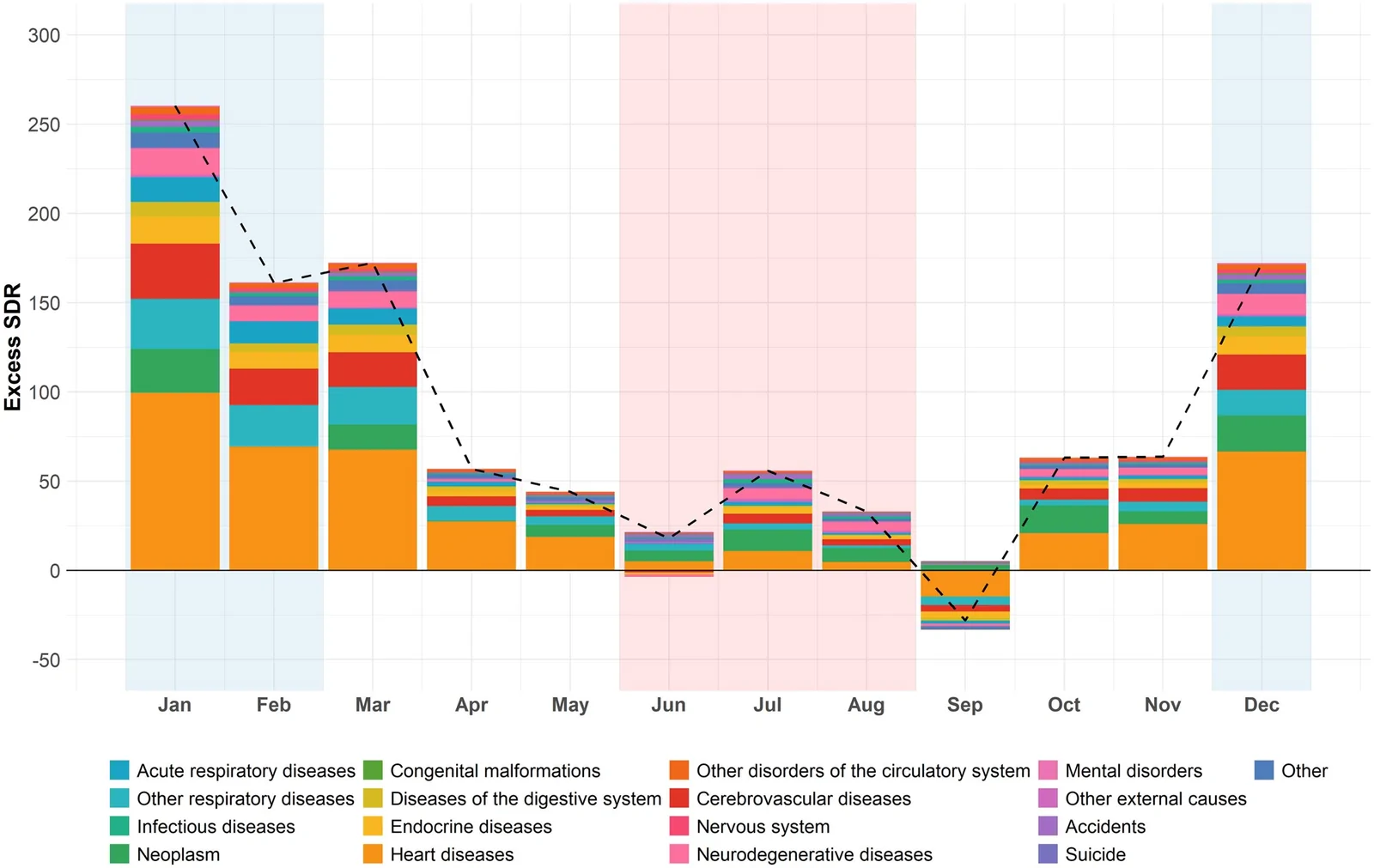
Excess mortality by month and cause of death, total Italian population (blue shadow: winter, red shadow: summer), average 2004–19. Note. Negative values for cause-specific contributions occasionally emerged during periods of low mortality and are likely due to unstable estimates caused by small death counts.

Analyzing excess mortality from all causes of death (black line in Fig. 2), we observed a clear seasonal pattern, with the strongest intra-annual fluctuations consistently occurring during the winter months (highlighted by the blue background shading). During the winter months, the dominant contributors were heart diseases, accounting for approximately 40% of total excess mortality (Fig. 2 and Table 1). Additional significant contributors included cerebrovascular diseases and respiratory diseases, particularly acute respiratory infections, which together contributed between 12% and 16% to the overall excess intra-annual mortality. Summer excess mortality was composed not only of heart mortality (20.6% on average) but also of neoplasms (26.4% on average), neurodegenerative diseases (6.5% in total, but 13% when considering only July and August, see Table S2 for month-specific results), and suicides (17% in June). However, since the total number of excess deaths during the summer months averaged only around 50 excess deaths, these percentage contributions correspond to relatively small absolute numbers. The analysis at the NUTS1 levels showed very similar patterns both in terms of seasonality and cause-of-death composition of excess mortality (Fig. S8).

**Table 1:** Excess mortality by month and cause of death, total Italian population, (blue shadow: winter, red shadow: summer), average 2004–19.

Causes of deaths	Winter			Summer			Annual		
	Total	Female	Male	Total	Female	Male	Total	Female	Male
All causes of death	593.4	522.5	709.6	106.6	102.1	108.6	1071.7	942.0	1286.4
Acute respiratory	5.4%	5.6%	5.0%	3.5%	4.0%	4.1%	4.7%	5%	4.0%
Other respiratory	11.0%	9.5%	12.8%	7.9%	6.5%	6.8%	10.2%	9%	11.3%
Infectious	1.2%	1.3%	1.2%	4.0%	4.4%	3.3%	1.5%	2%	1.4%
Neoplasm	7.5%	6.5%	8.8%	24.2%	19.8%	21.8%	11.5%	10%	12.1%
Congenital	0.2%	0.3%	0.3%	1.0%	1.1%	−0.1%	0.5%	1%	0.4%
Digestive system	3.1%	2.8%	3.4%	1.5%	1.9%	2.5%	3.0%	3%	3.3%
Endocrine	5.8%	6.2%	5.4%	4.2%	6.4%	6.3%	5.1%	6%	4.9%
Heart	39.7%	39.7%	38.7%	19.3%	20.5%	19.7%	37.0%	37%	37.0%
Other circulatory	1.6%	1.5%	1.8%	0.6%	1.1%	1.0%	1.8%	2%	2.1%
Cerebrovascular	12.0%	12.6%	11.2%	7.1%	10.2%	11.2%	11.0%	11%	10.4%
Nervous system	1.2%	1.3%	1.2%	1.4%	1.3%	0.6%	1.2%	1%	1.1%
Neurodegenerative	5.9%	6.6%	4.9%	9.3%	12.9%	9.5%	5.7%	7%	4.9%
Mental disorders	0.3%	0.3%	0.4%	0.8%	0.7%	−0.1%	0.4%	0%	0.3%
Other external causes	0.5%	0.6%	0.5%	3.5%	1.9%	3.2%	0.9%	1%	1.0%
Accidents	1.2%	1.8%	0.8%	5.7%	3.2%	5.4%	1.7%	2%	1.9%
Suicide	0.1%	0.1%	0.2%	3.4%	1.3%	1.0%	0.9%	1%	0.8%
Other	3.2%	3.2%	3.3%	2.7%	2.9%	3.7%	2.8%	3%	3.2%

### Relative impact of causes of death on excess all-cause mortality

**Figure 3:**
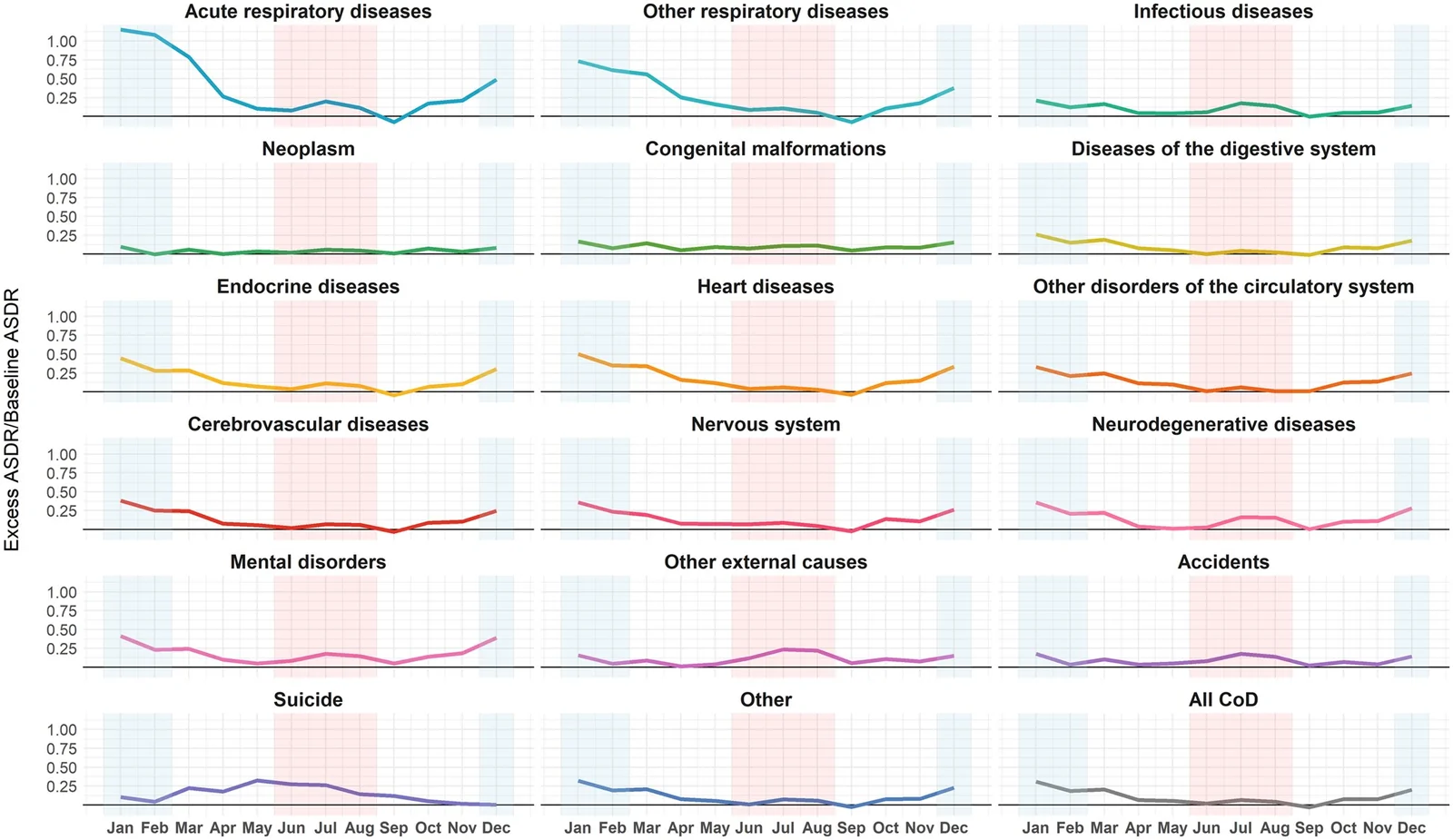
Relative ratio of observed SDR and baseline SDR by month and cause of death (blue shadow: winter, red shadow: summer), total Italian population, average 2004–19.

To evaluate the relative burden of intra-annual mortality fluctuations, we examined the relative ratio between cause-specific observed mortality and the corresponding baseline mortality levels. This ratio captures the relative impact of intra-annual mortality excess deaths for each cause of death. Respiratory diseases, especially acute respiratory infections, stood out with a ratio substantially above 0, reaching, on average, 90% more than the baseline level during January and February (Fig. 3). This finding indicates that, although heart diseases remain the largest contributors to overall excess mortality in absolute terms, respiratory diseases are the most sensitive to seasonal fluctuations, particularly during winter.

### Sex differences in the cause-specific contributions

Furthermore, analyzing the results by sex (Fig. S3 and Table 1), we observed a larger impact of intra-annual mortality on male mortality both during winter months and summer months (respectively, 710 excess deaths and 110 excess deaths) than in the female population (520 and 100), reflecting the higher underlying mortality level in men. We also found similar patterns in excess mortality by causes of death during winter, but not during summer. In fact, in the female population, we observed a larger contribution of neurodegenerative diseases, while in the male population, a higher contribution of accidents and other external causes. Looking at the results for the relative impacts, we found that females had a relatively higher burden of mortality by respiratory disease than males during the winter months (Fig. S4).

### Trends over time in intra-annual mortality fluctuations by causes of death

In Italy, all-cause mortality declined between 2004 and 2019, though trends varied by cause of death. Mortality from infectious diseases, nervous system and neurodegenerative conditions, and mental disorders increased, while respiratory and endocrine diseases, suicide, and other external causes remained stable. In contrast, neoplasms, heart disease, cerebrovascular disease, and accidents showed clear declines (Fig. S1). Both the absolute and the relative impact of different causes of death on excess mortality remained largely unchanged over time (Figs. S5 and S6). This suggests that the seasonal burden has not diminished proportionally and the relative impact may even be rising for specific causes, such as neurodegenerative (from 3% in 2004 to 8% in 2019) and respiratory diseases (from 4% in 2004 to 7% in 2019), pointing to an increasing vulnerability of certain subpopulations to seasonal stressors, which may act in combination with a possible increase in the intensity or frequency of these stressors over time.

## Discussion

### Main findings

This study assesses cause-specific patterns of seasonal mortality and their contribution to overall seasonal mortality in Italy between 2004 and 2019 in a consistent and comparable manner. The results revealed that excess mortality varies by cause of death and that particular cause exhibit pronounced seasonal patterns.

As expected, heart and respiratory diseases emerged as the main drivers of winter excess mortality (40% and 16%, respectively). While heart diseases accounted for the largest proportion of absolute excess deaths, respiratory mortality displayed the highest relative deviations from baseline levels. Importantly, these seasonal peaks remained persistent over time, even as overall baseline mortality declined, indicating a growing or unmitigated seasonal burden for specific causes of death. We found that excess heart mortality not only shaped the total excess mortality but also had one of the highest winter excess mortality levels, while only minor excess mortality occurred in summer.

Despite the seasonal impact being lower during summer months compared to winter months, we found different contributing causes of death compared to winter. In fact, neoplasms, accidents, and neurodegenerative diseases played a more prominent role in excess mortality during summer. Moreover, the male population was more affected by the intra-annual mortality fluctuations, considering all-cause mortality and each specific cause of death; nevertheless, neurodegenerative conditions were more relevant among females, especially in summer.

Furthermore, although we observed a general decline in baseline mortality levels across most causes of death over the study period, the relative excess mortality remained stable for almost all causes.

### Explanation of the findings

The observed impact of intra-annual fluctuations on mortality in Italy was driven by heart and respiratory diseases. The observed winter peaks in heart (absolute terms) and respiratory mortality (relative terms) were consistent with a large body of literature documenting the adverse effects of cold temperatures and seasonal infections on cardiorespiratory outcomes [6, 27, 28]. These effects are well-established and are often linked to increased incidence of influenza-like illnesses, reduced indoor air quality, and physiological stress induced by cold exposure.

We found that the impact of summer on mortality was considerably smaller than that of winter. While heart diseases remained important contributors, other causes, such as neoplasms, neurodegenerative diseases, and suicides, also played a role. Although summer excess mortality is markedly lower than in winter, it likely reflects partly distinct underlying mechanisms. Winter peaks are more closely associated with respiratory infections, poorer air quality, and cold-related physiological stress, whereas summer excess is more plausibly linked to heat exposure and related vulnerabilities. These seasonal differences likely arise from a mix of behavioral, biological, and structural risk factors, including exposure conditions and access to healthcare [1, 29, 30]. Such vulnerabilities may also interact with underlying disparities in the leading causes of death. For example, neurocognitive disorders, which are more common among older women [31], can increase their susceptibility to heat-related mortality [31]. It is also important to note that our analysis does not cover the major 2003 heatwave (due to the unavailability of data), which caused more than 20 000 excess deaths in Italy [32], nor the recent heatwaves in 2022 [33] and 2023 [34]. Within the period studied, however, the observed summer mortality patterns suggest that typical seasonal heat and low-intensity heatwaves have not substantially affected excess mortality in Italy.

Of particular note is the finding that, despite steady improvements in overall mortality over the analyzed period in Italy [25], the relative burden of intra-annual mortality fluctuations has remained persistent, similar to other European countries [5]. Moreover, the composition of absolute excess mortality by cause has remained stable over time, suggesting that the seasonal burden has not diminished in step with overall mortality improvements. It may even be increasing for certain causes, such as neurodegenerative and respiratory diseases, pointing to growing vulnerability to seasonal stressors in specific subpopulations. In contrast, relative excess mortality showed little change over time, likely because observed and baseline mortality followed similar temporal patterns.

A closer look at age-specific patterns reveals who is the most affected. Although our analysis relies on a standardized measure of mortality, which limits direct interpretation of age-specific contributions, we further examined cause- and age-specific death rates. As expected, mainly the oldest age groups (75–89 and 90+) exhibited a clear seasonal pattern in mortality, particularly for respiratory and cardiovascular mortality. In contrast, younger age groups showed relatively stable mortality throughout the year, with minimal seasonal fluctuations. These findings suggest that intra-annual excess mortality, especially during winter months, is largely driven by older individuals, highlighting their higher vulnerability to seasonal and environmental stressors.

The patterns observed in Italy likely reflect broader dynamics seen across Southern and Central Europe. In our previous work [5], we found that seasonal fluctuations in mortality and their impact on life expectancy share common features across European countries, particularly those with similar climatic conditions and population age structures. However, the contribution of specific causes of death to these seasonal patterns can differ markedly between countries. For example, multi-country research has shown that cold weather tends to increase cardiovascular and respiratory mortality, especially from heart failure [35, 36], yet the relative weight of these causes varies depending on national profiles. Factors such as population age structure, health system capacity, and housing conditions (e.g. thermal insulation, air conditioning) can shape both the magnitude and composition of seasonal excess mortality. This underscores that while the seasonal mechanisms we identify in Italy may be relevant elsewhere, targeted interventions must account for country-specific cause-of-death patterns and vulnerabilities.

### Strengths and limitations of the study

A key strength of this study is our methodological approach, which allowed for a standardized comparison of intra-annual fluctuations across time and causes of death. This approach allows the estimation of the actual impact of intra-annual mortality fluctuations on observed cause-specific mortality trends. Another added value is the use of monthly, cause-specific population-level mortality data from official statistics produced by the Italian National Statistical Institute (ISTAT).

Nevertheless, several limitations must be acknowledged. First, the analysis assumes intra-month homogeneity in mortality patterns, which may overlook extreme short-term events such as heatwaves concentrated within a few days and compensated by the following harvesting effect. Second, while we attempted to mitigate the influence of long-term trends and counterbalance seasonal effects by selecting a consistent reference mortality level (validated through sensitivity analyses in previous work [5, 26]), our choice of baseline is suboptimal when the focus is on isolating temperature-attributable mortality. In particular, defining the baseline as the 3 months with the lowest SDR within each year implies that it reflects mortality observed under specific seasonal conditions (often summer). As a result, the baseline serves as a within-year reference point for relative comparisons, but may not be directly comparable across seasons. Although alternative baselines may be defined under different assumptions, additional analyses using the median-mortality month produced comparable seasonal patterns, albeit with slightly greater variability, indicating that baseline choice mainly affects precision rather than substantive conclusions (Appendix C). Third, although mortality data in Italy are of high quality [37], the high level of detail required for our analysis (monthly data by cause of death) limited our ability to examine smaller, more specific causes, such as heatstroke or dehydration, which may have important implications for interpreting seasonal mortality patterns. Fourth, our results may partially reflect underlying differences in the distribution of causes of death between men and women. Since seasonal mortality is driven primarily by specific causes, sex differences in the prevalence of these causes contribute to observed patterns.

## Conclusion

Our findings underscore that, seasonal vulnerabilities, particularly for cardiovascular and respiratory diseases in winter, continue to have a strong influence on short-term mortality risks, especially among the oldest age groups. The results highlight the importance of taking seasonal dynamics into account when planning public health measures. Interventions such as targeted wintertime prevention campaigns for respiratory and cardiovascular conditions, or summer safety efforts focused on accidents and heat-related risks (heatstroke, dehydration), could help reduce avoidable deaths, particularly for the elderly population [14]. This challenge will become even more relevant in the coming years, as climate change associated with extreme temperatures is likely to increase the health risks in rapidly ageing societies. Reliable evidence about specific subpopulations at risk is also a precondition for addressing emerging health threats and healthcare system planning [38, 39].

## Supplementary Material

ckag165_Supplementary_Data

## Data Availability

Monthly death counts by causes of death are available upon request to the ISTAT contact centre, while population exposures are openly available on the National Italian Statistical Institute (ISTAT) website (https://www.istat.it/en/). Key points Seasonal mortality patterns are known in Europe, with peaks in winter (flu-related) and smaller peaks in summer (heatwaves), but no comprehensive knowledge exists on how different causes of death shape these fluctuations. This study applies a novel framework to quantify, in a systematic, transparent and comparable manner, the absolute and relative contributions of 17 cause-of-death groups to seasonal mortality in Italy (2004–19) We found that winter mortality, due to excess mortality from heart and respiratory diseases, is the key driver of excess seasonal mortality, highlighting persistent vulnerabilities despite overall mortality improvements. Seasonal mortality remains a significant, cause-specific public health challenge; public health policy should prioritize targeted, cause- and season-specific prevention strategies to reduce excess mortality and strengthen resilience to short-term risk factors.
