# Supplementary material for: Analysis of intra-annual mortality fluctuations by cause of death in Italy, 2004–19
Source: Eur J Public Health. 2026 Sep 22;36(5):ckag165. doi: 10.1093/eurpub/ckag165 (PMC13596777; doi:10.1093/eurpub/ckag165)
Supplement: Supplementary file 1 [file ckag165_supplementary_data.zip › ejph-2025-10-om-0844-File005.docx]

**Supplementary Material**

**Analysis of intra-annual mortality fluctuations by causes of death in Italy, 2004-2019**

**European Journal of Public Health**

Isabella Marinetti^1,2*^, Dmitri A. Jdanov, France Meslé, Domantas Jasilionis, Fanny Janssen

^1^Max Planck Institute for Demographic Research, Rostock, Germany

^2^Population Research Centre, Faculty of Spatial Sciences, University of Groningen, Groningen, The Netherlands

*Corresponding author: Isabella Marinetti [marinetti@demogr.mpg.de](mailto:marinetti@demogr.mpg.de)

**Appendix A: Cause-of-Death Data**

We followed the Human Causes of Death short-list to determine the causes of death groups, adding also neurodegenerative diseases (relevant in an ageing population, such as the Italian one) and differentiated external causes in accidents, suicides and other external causes.

Ill-defined causes of death (ca. 1.8% of deaths on average between 2004-2019), ICD-10 codes: R00-R94, R96-R99, were proportionally redistributed to the other causes of death. We define seasons based on calendar years (Winter: January, February, December; Spring: March, April, May; Summer: June, July, August; Autumn: September, October, November). The analyses were carried out for each year and for the overall period average. Furthermore, we excluded deaths due to drowning (ICD-10 codes W69-W74) in the Insular region, as data from the statistical office also included casualties from shipwrecks that occurred in Italian territorial waters, which are not necessarily linked to resident populations, but rather to migrant fatalities (approximately 0.01% of the total deaths between 2004-2019).

**Appendix B: Analysis by regions (NUTS1)**

The analysis at the NUTS1 level showed similar results. All territories followed a similar seasonal pattern compared to the whole country; however, with different magnitudes, both in terms of seasonality and cause-of-death composition of excess mortality.

See figures S7 and S8.

**Appendix C: Different baseline method**

Our choice to define baseline as the months with the lowest mortality each year is intentionally designed to avoid attributing excess mortality to a single external risk factor, such as temperature. A baseline based strictly on temperature may risk oversimplifying the mechanisms behind excess mortality and could limit comparability across different causes of death.

To evaluate the robustness of our methodological choice, we used an alternative baseline defined as the median month in terms of the number of deaths. This approach provides a more central reference point in the annual mortality distribution. The results indicate that the main patterns of excess mortality remain consistent with our original specification. However, as expected, estimates based on the median month exhibit slightly greater random variability, particularly for causes of death with small case numbers in certain regions or years. This suggests that while different baseline choices can affect the precision of estimates, they do not substantially alter our core findings regarding the seasonality of mortality (Figure S9).

**Appendix D: Confidence Intervals**

Assuming Poisson-distributed deaths, the variance of the excess mortality by cause of death was approximated as in (Keyfitz, 1966):

$Var\left( {SDR}_{c,t}^{excess, corr} \right)=\sum_{x} w_{x}^{2}\cdot Var\left( \frac{m_{x,c,t}^{observed}}{Pop_{x,t}}+\frac{m_{x,c,t}^{ideal}}{Pop_{x,t}} \right)$ (1)

Where $w_{x}=\frac{P_{x}^{s}}{\sum_{x} P_{x}^{s}}$ are the population weights and $P_{x}^{s}$ is the European Standard Population 2013 at age *x*.

The standard error of the excess mortality was then computed as:

$SE_{c,t}=\sqrt{Var\left( {SDR}_{c,t}^{excess, corr} \right)}$ (2)

and 95% confidence intervals were constructed using a normal approximation:

${SDR}_{c,t}^{excess, corr}\pm1.96 \cdot SE_{c,t}$ (3)

Similarly, the confidence intervals were computed for all-cause excess mortality, substituting ${SDR}_{c,t}^{excess, corr}$ with ${SDR}_{tot,t}^{excess}$ and using all-cause mortality rates instead of cause-specific. The confidence intervals were calculated independently by month.

Below is the confidence interval table for each cause of death and month.

***Reference***

*Keyfitz, N. (1966). Sampling Variance of Standardized Mortality Rates. Human Biology, 38(3), 309–317.*

**Supplementary Tables**

**Table S1.** List of ICD-10 codes used in the analysis and their percentage deaths by sex, 2004-2019 average

| Causes of death | ICD-10 code | Deaths 2004-2019 (%) | | |
| --- | --- | --- | --- | --- |
|  |  | **Tot** | **Male** | **Female** |
| Acute respiratory diseases | J00-J22, U04-U07 | 1.8% | 1.7% | 1.9% |
| Other respiratory diseases | J30-J98 | 5.5% | 6.4% | 4.5% |
| Infectious diseases | A00-A99, B00-B99 | 1.9% | 1.9% | 1.9% |
| Neoplasm | C00-C99, D00-D48 | 29.6% | 34.3% | 25.3% |
| Congenital malformations | P00-P99, Q00-Q99, R95 | 0.4% | 0.5% | 0.4% |
| Diseases of the digestive system | K00-K63, K65-K92 | 3.9% | 4.0% | 3.9% |
| Endocrine diseases | E00-E88 | 4.5% | 3.9% | 5.0% |
| Heart diseases | I00-I51 | 26.5% | 24.2% | 28.7% |
| Other disorders of the circulatory system | I70-I99, K64 | 1.6% | 1.7% | 1.5% |
| Cerebrovascular diseases | G45, I60-I69 | 10.3% | 8.3% | 12.1% |
| Nervous system | G00-G19, G24-G29, G33-G44, G47-G93 | 1.1% | 1.1% | 1.0% |
| Neurodegenerative Diseases | F00-F03, G20-G23, G30-G32 | 5.4% | 4.0% | 6.8% |
| Mental disorders | F04-F99 | 0.3% | 0.3% | 0.2% |
| Other external causes | W00-W68, W75-W99, Y00-Y99 | 1.1% | 1.2% | 1.0% |
| Accident | V00-V99, X00-X59 | 2.3% | 2.6% | 2.0% |
| Suicide | X60-X84 | 0.7% | 1.1% | 0.3% |
| Other | D50-D89, E89-E90, H00-H95, L00-L98, M00-M99, N00-N99, O00-O99, S00-S99, T00-T98, U00-U49, U82-U85, Z00-Z99 | 3.2% | 2.8% | 3.6% |

**Table S2.** Excess mortality by month and cause of death, total Italian population, (blue shadow: winter, red shadow: summer, darker shadow: high contributions), 2004-2019 average

| Month | Jan | Feb | Mar | Apr | May | Jun | Jul | Aug | Sept | Oct | Nov | Dec | TOT |
| --- | --- | --- | --- | --- | --- | --- | --- | --- | --- | --- | --- | --- | --- |
| All Causes of Death | 260.3 | 160.9 | 172.3 | 56.8 | 44.0 | 17.8 | 55.9 | 32.9 | -28.1 | 63.1 | 63.6 | 172.1 | 1071.6 |
| Acute respiratory | 5.3% | 7.7% | 5.2% | 4.8% | 2.2% | 2.9% | 3.8% | 3.3% | 5.5% | 2.7% | 2.9% | 3.3% | 4.7% |
| Other respiratory | 10.8% | 14.4% | 12.2% | 14.6% | 10.8% | 19.4% | 6.3% | 4.4% | 16.9% | 5.4% | 8.6% | 8.3% | 10.5% |
| Infectious | 1.2% | 1.1% | 1.4% | 1.1% | 1.1% | 2.8% | 4.1% | 4.5% | 0.0% | 0.9% | 1.0% | 1.2% | 1.5% |
| Neoplasm | 9.4% | -0.2% | 8.2% | 0.5% | 15.1% | 34.2% | 21.4% | 23.4% | -10.6% | 24.5% | 11.0% | 11.8% | 10.9% |
| Congenital | 0.2% | 0.2% | 0.3% | 0.4% | 0.6% | 2.8% | 0.6% | 0.8% | -2.0% | 0.4% | 0.5% | 0.3% | 0.4% |
| Digestive system | 3.1% | 2.9% | 3.4% | 3.7% | 3.0% | -0.3% | 1.9% | 1.6% | 5.9% | 4.0% | 3.4% | 3.3% | 3.0% |
| Endocrine | 5.8% | 5.8% | 5.6% | 6.1% | 4.4% | -5.3% | 6.2% | 6.0% | 12.5% | 3.3% | 4.7% | 5.9% | 5.2% |
| Heart | 38.2% | 43.1% | 39.2% | 48.1% | 42.6% | 28.0% | 19.4% | 14.4% | 52.3% | 32.9% | 40.9% | 38.6% | 37.5% |
| Other circulatory | 1.6% | 1.6% | 1.8% | 2.1% | 2.3% | -1.1% | 1.2% | 0.5% | 0.8% | 2.3% | 2.4% | 1.7% | 1.7% |
| Cerebrovascular | 11.9% | 12.7% | 11.3% | 9.6% | 8.3% | -7.1% | 9.5% | 10.5% | 13.0% | 9.8% | 11.8% | 11.4% | 10.9% |
| Nervous system | 1.2% | 1.2% | 0.9% | 1.0% | 1.2% | 3.3% | 1.2% | 0.8% | -0.7% | 1.6% | 1.2% | 1.3% | 1.3% |
| Neurodegenerative | 5.8% | 5.3% | 5.1% | 2.5% | 0.7% | -6.7% | 10.8% | 15.3% | 5.3% | 6.0% | 6.1% | 6.6% | 5.7% |
| Mental disorders | 0.3% | 0.3% | 0.3% | 0.3% | 0.2% | 2.0% | 0.6% | 0.6% | -1.1% | 0.3% | 0.5% | 0.4% | 0.4% |
| Other external causes | 0.5% | 0.3% | 0.4% | 0.1% | 0.5% | 4.7% | 2.9% | 3.7% | -2.0% | 1.1% | 0.9% | 0.7% | 0.9% |
| Accidents | 1.3% | 0.4% | 1.1% | 0.6% | 1.8% | 8.4% | 5.1% | 5.3% | -0.9% | 1.7% | 1.0% | 1.6% | 1.7% |
| Suicide | 0.2% | 0.1% | 0.7% | 1.4% | 2.9% | 11.4% | 2.0% | 1.5% | -1.5% | 0.4% | 0.1% | 0.0% | 0.8% |
| Other | 3.1% | 3.0% | 3.0% | 3.0% | 2.3% | 0.6% | 3.0% | 3.2% | 6.6% | 2.7% | 2.8% | 3.4% | 2.9% |

**Table S3.** Excess mortality by cause of death and for all-cause mortality by month, total Italian population, median mortality month baseline, 2004-2019 average

| Cause of Death | January | February | March | April | May | June | July | August | September | October | November | December |
| --- | --- | --- | --- | --- | --- | --- | --- | --- | --- | --- | --- | --- |
| Acute respiratory diseases | 15.4 | 18.4 | 10.9 | 1 | 5.1 | 0.1 | 1.5 | 1.2 | -1.1 | 2 | -15.4 | 5.4 |
| Other respiratory diseases | 29.5 | 32.8 | 24.5 | -4.2 | 5.5 | -0.4 | -1.4 | 1.5 | -3.1 | -86.4 | -9.4 | 13.6 |
| Infectious diseases | 2.8 | 0.7 | 2.2 | 7.4 | -0.6 | 1.2 | 11.8 | 2.2 | -0.4 | -18.6 | 2.1 | 1.6 |
| Neoplasm | 21.3 | -25.7 | 9.3 | 44.5 | 6.1 | 2.4 | 17.7 | 8.1 | -2.5 | 349.1 | -41.9 | 19.4 |
| Congenital malformations | 0.3 | -0.2 | 0.3 | -0.8 | -1.1 | 0.5 | -0.4 | 0.4 | -0.1 | 1.2 | -7.8 | 0.4 |
| Diseases of the digestive system | 8.2 | 4.3 | 6.2 | -0.9 | -6.7 | -3 | -2.1 | 0.4 | -0.9 | -20 | 1.2 | 5.6 |
| Endocrine diseases | 15.9 | 10.7 | 10.3 | 0.3 | -3.3 | 0.2 | 5.3 | 2.2 | -1.8 | -40.2 | -5.4 | 10.9 |
| Heart diseases | 100.5 | 83.4 | 69.5 | -19.7 | 1 | 1.9 | -29.2 | 4.5 | -11.4 | -233 | 115.6 | 67.7 |
| Other disorders of the circulatory system | 3.7 | 2.2 | 2.7 | 4.3 | 0.2 | -0.7 | -0.5 | -0.2 | -0.5 | 3.5 | 39 | 2.5 |
| Cerebrovascular diseases | 32.7 | 24 | 20.1 | 9.9 | 17.7 | 7 | 4.6 | 2.5 | -3.3 | 21.9 | 18 | 20.7 |
| Nervous system | 3 | 1.9 | 1.2 | 3.5 | -0.2 | 2.6 | -2 | 0.2 | -0.4 | 4.6 | -16.7 | 2.2 |
| Neurodegenerative diseases | 14.8 | 7.3 | 7.8 | 18.8 | 14.5 | 0.6 | 30.6 | 6 | -1.3 | 80.7 | 6.6 | 12.5 |
| Mental disorders | 0.8 | 0.2 | 0.4 | -1.6 | -0.3 | 0.2 | 0.3 | 0 | -0.1 | -0.6 | 0.8 | 0.8 |
| Other external causes | 0.7 | -1.1 | 0 | -3.6 | 0.1 | -0.9 | 4.3 | 1 | -0.1 | 15.2 | -0.3 | 0.9 |
| Accidents | 2.4 | -2.2 | 0.6 | 1.7 | 2.7 | 5.8 | 8.3 | 1.7 | -0.4 | -4 | -2.6 | 2.3 |
| Suicide | -0.1 | -1.1 | 0.9 | -4.7 | 4.2 | 0.7 | 6 | 0 | 0 | -7.6 | -23 | -0.9 |
| Other | 8.4 | 5.1 | 5.4 | 1.1 | -0.9 | -0.4 | 1.1 | 1.2 | -0.9 | -4.9 | 2.9 | 6.6 |
| All-cause | 202.1 | 98.8 | 112.3 | -4.6 | -14.8 | -45.6 | -6.1 | -26.5 | -88.6 | 3.5 | 4.8 | 110.6 |

**Table S4.** Excess mortality estimates with 95% confidence intervals by cause of death and for all-cause mortality by month, total Italian population, 2004-2019 average

| Cause of Death | January | February | March | April | May | June | July | August | September | October | November | December |
| --- | --- | --- | --- | --- | --- | --- | --- | --- | --- | --- | --- | --- |
| Acute respiratory diseases | 13.9 (13.4; 14.4) | 12.2 (11.7; 12.6) | 8.9 (8.5; 9.3) | 2.6 (2.3; 2.9) | 1 (0.7; 1.3) | 0.3 (-0.5; 1.1) | 2 (1.7; 2.3) | 1.1 (0.9; 1.4) | -1.3 (-1.9; -0.6) | 1.7 (1.4; 2.1) | 2.1 (1.8; 2.5) | 5.6 (5.2; 6) |
| Other respiratory diseases | 28.3 (27.5; 29.1) | 22.9 (22.2; 23.7) | 21 (20.3; 21.8) | 8.2 (7.6; 8.8) | 4.9 (4.4; 5.5) | 3.2 (1.7; 4.6) | 3.5 (3; 4) | 1.6 (1.1; 2) | -6.7 (-7.9; -5.5) | 3.6 (3; 4.2) | 5.9 (5.3; 6.5) | 14.2 (13.4; 14.9) |
| Infectious diseases | 3.2 (2.8; 3.6) | 1.8 (1.4; 2.2) | 2.4 (1.9; 2.8) | 0.6 (0.2; 0.9) | 0.5 (0.2; 0.8) | 0.5 (-0.4; 1.4) | 2.2 (1.9; 2.6) | 1.6 (1.3; 1.9) | 0.1 (-0.6; 0.8) | 0.6 (0.2; 0.9) | 0.7 (0.3; 1) | 2 (1.6; 2.4) |
| Neoplasm | 24.5 (22.8; 26.3) | -0.5 (-2.1; 1.2) | 14.3 (12.6; 16) | 0.3 (-1.1; 1.8) | 6.9 (5.6; 8.3) | 6.9 (3.2; 10.5) | 12 (10.7; 13.4) | 7.9 (6.9; 9) | 8.2 (5.2; 11.2) | 15.9 (14.4; 17.4) | 7.4 (5.9; 8.9) | 20.2 (18.5; 21.8) |
| Congenital malformations | 0.6 (0.4; 0.9) | 0.3 (0.1; 0.5) | 0.5 (0.3; 0.8) | 0.2 (0; 0.4) | 0.3 (0.1; 0.5) | 0.7 (0.2; 1.2) | 0.3 (0.2; 0.5) | 0.3 (0.1; 0.4) | 0.9 (0.5; 1.3) | 0.3 (0.1; 0.5) | 0.3 (0.1; 0.5) | 0.6 (0.3; 0.8) |
| Diseases of the digestive system | 8.3 (7.6; 8.9) | 4.8 (4.1; 5.4) | 6 (5.4; 6.7) | 2.1 (1.6; 2.6) | 1.4 (0.9; 1.9) | -0.4 (-1.7; 1) | 1.1 (0.7; 1.6) | 0.5 (0.2; 0.9) | -1.5 (-2.6; -0.4) | 2.5 (2; 3.1) | 2.2 (1.6; 2.7) | 5.7 (5; 6.3) |
| Endocrine diseases | 15.3 (14.6; 16) | 9.2 (8.6; 9.9) | 9.5 (8.9; 10.2) | 3.3 (2.8; 3.9) | 2 (1.5; 2.5) | -1.3 (-2.7; 0.1) | 3.4 (2.9; 3.9) | 2.1 (1.7; 2.5) | -4.1 (-5.2; -2.9) | 2.1 (1.5; 2.6) | 3.1 (2.5; 3.6) | 10.1 (9.5; 10.8) |
| Heart diseases | 100.6 (98.9; 102.3) | 69.1 (67.5; 70.7) | 67.8 (66.2; 69.5) | 27.1 (25.8; 28.5) | 19.5 (18.3; 20.8) | 3.7 (0.3; 7.1) | 10.6 (9.4; 11.8) | 5 (4.1; 6) | -19 (-21.8; -16.1) | 21.1 (19.8; 22.5) | 26.5 (25.1; 27.9) | 66.4 (64.7; 68) |
| Other disorders of the circulatory system | 4.2 (3.8; 4.6) | 2.7 (2.3; 3.1) | 3.1 (2.7; 3.5) | 1.2 (0.9; 1.6) | 1 (0.7; 1.4) | -0.4 (-1.2; 0.5) | 0.6 (0.3; 0.9) | 0.1 (-0.1; 0.4) | 0.3 (-0.4; 1) | 1.4 (1.1; 1.8) | 1.5 (1.2; 1.9) | 3 (2.6; 3.4) |
| Cerebrovascular diseases | 31.4 (30.3; 32.5) | 20.4 (19.4; 21.4) | 19.8 (18.8; 20.9) | 5.4 (4.6; 6.3) | 4 (3.2; 4.8) | -1.3 (-3.5; 0.8) | 5 (4.3; 5.8) | 3.8 (3.2; 4.4) | -4.1 (-5.9; -2.2) | 6.4 (5.5; 7.2) | 7.7 (6.9; 8.6) | 19.9 (18.9; 20.9) |
| Nervous system | 3.1 (2.8; 3.5) | 2 (1.6; 2.3) | 1.6 (1.3; 1.9) | 0.6 (0.3; 0.8) | 0.5 (0.3; 0.8) | 0.4 (-0.2; 1.1) | 0.6 (0.4; 0.9) | 0.3 (0.1; 0.5) | 0 (-0.5; 0.5) | 1 (0.7; 1.3) | 0.8 (0.5; 1.1) | 2.2 (1.8; 2.5) |
| Neurodegenerative diseases | 15.4 (14.7; 16.2) | 8.4 (7.7; 9.1) | 8.7 (8; 9.4) | 1.4 (0.8; 2) | 0.5 (-0.1; 1) | -2.7 (-4.2; -1.1) | 5.8 (5.3; 6.4) | 5.3 (4.8; 5.7) | -0.1 (-1.3; 1.1) | 3.8 (3.2; 4.4) | 4.1 (3.5; 4.7) | 11.3 (10.6; 12) |
| Mental disorders | 0.8 (0.7; 1) | 0.5 (0.3; 0.6) | 0.5 (0.3; 0.6) | 0.2 (0; 0.3) | 0.1 (0; 0.2) | 0.5 (0.1; 0.8) | 0.3 (0.2; 0.4) | 0.2 (0.1; 0.3) | 0.3 (0.1; 0.6) | 0.2 (0.1; 0.4) | 0.3 (0.2; 0.5) | 0.8 (0.6; 0.9) |
| Other external causes | 1.4 (1; 1.7) | 0.4 (0.1; 0.7) | 0.7 (0.4; 1.1) | 0.1 (-0.2; 0.4) | 0.3 (0; 0.5) | 1.4 (0.7; 2.2) | 1.7 (1.4; 1.9) | 1.3 (1; 1.5) | 0.2 (-0.3; 0.8) | 0.8 (0.5; 1.1) | 0.6 (0.3; 0.9) | 1.3 (0.9; 1.6) |
| Accidents | 3.4 (2.9; 3.9) | 0.7 (0.2; 1.1) | 1.9 (1.4; 2.4) | 0.6 (0.2; 1) | 0.9 (0.5; 1.3) | 1.6 (0.6; 2.7) | 2.9 (2.5; 3.3) | 1.7 (1.4; 2) | 0.1 (-0.7; 1) | 1.2 (0.8; 1.6) | 0.7 (0.3; 1.1) | 2.7 (2.2; 3.2) |
| Suicide | 0.5 (0.3; 0.8) | 0.2 (0; 0.4) | 1.2 (0.9; 1.4) | 0.8 (0.6; 1) | 1.4 (1.1; 1.6) | 2.6 (2; 3.2) | 1.1 (0.9; 1.3) | 0.5 (0.4; 0.7) | 1.1 (0.6; 1.5) | 0.2 (0; 0.5) | 0.1 (-0.1; 0.3) | 0 (-0.2; 0.3) |
| Other | 8.2 (7.6; 8.8) | 4.8 (4.2; 5.3) | 5.3 (4.7; 5.8) | 1.7 (1.2; 2.2) | 1.1 (0.7; 1.5) | -0.3 (-1.5; 0.9) | 1.6 (1.2; 2.1) | 1.1 (0.8; 1.5) | -2.1 (-3.1; -1.2) | 1.7 (1.2; 2.2) | 1.8 (1.3; 2.3) | 5.7 (5.1; 6.3) |
| All-cause | 263.1 (259.6; 266.6) | 159.8 (156.3; 163.2) | 173.3 (169.9; 176.7) | 56.4 (53.1; 59.7) | 46.2 (42.9; 49.5) | 15.4 (12.1; 18.7) | 54.9 (51.6; 58.2) | 34.5 (31.2; 37.8) | -27.6 (-30.8; -24.4) | 64.5 (61.2; 67.9) | 65.8 (62.5; 69.1) | 171.6 (168.1; 175) |
